# MMS21/HPY2 and SIZ1, Two Arabidopsis SUMO E3 Ligases, Have Distinct Functions in Development

**DOI:** 10.1371/journal.pone.0046897

**Published:** 2012-10-08

**Authors:** Takashi Ishida, Mika Yoshimura, Kenji Miura, Keiko Sugimoto

**Affiliations:** 1 RIKEN Plant Science Center, Tsurumi, Yokohama, Kanagawa, Japan; 2 Graduate School of Biological Sciences, Nara Institute of Science and Technology, Ikoma, Nara, Japan; 3 Faculty of Life and Environmental Sciences, University of Tsukuba, Tsukuba, Japan; University of Iowa, United States of America

## Abstract

The small ubiquitin related modifier (SUMO)-mediated posttranslational protein modification is widely conserved among eukaryotes. Similar to ubiquitination, SUMO modifications are attached to the substrate protein through three reaction steps by the E1, E2 and E3 enzymes. To date, multiple families of SUMO E3 ligases have been reported in yeast and animals, but only two types of E3 ligases have been identified in Arabidopsis: SAP and Miz 1 (SIZ1) and Methyl Methanesulfonate-Sensitivity protein 21 (MMS21)/HIGH PLOIDY 2 (HPY2), hereafter referred to as HPY2. Both proteins possess characteristic motifs termed Siz/PIAS RING (SP-RING) domains, and these motifs are conserved throughout the plant kingdom. Previous studies have shown that loss-of-function mutations in HPY2 or SIZ1 cause dwarf phenotypes and that the phenotype of *siz1-2* is caused by the accumulation of salicylic acid (SA). However, we demonstrate here that the phenotype of *hpy2-1* does not depend on SA accumulation. Consistently, the expression of *SIZ1* driven by the *HPY2* promoter does not complement the *hpy2-1* phenotypes, indicating that they are not functional homologs. Lastly, we show that the *siz1-2* and *hpy2-1* double mutant results in embryonic lethality, supporting the hypothesis that they have non-overlapping roles during embryogenesis. Together, these results suggest that SIZ1 and HPY2 function independently and that their combined SUMOylation is essential for plant development.

## Introduction

Small ubiquitin-related modifier (SUMO) is a member of the ubiquitin-like small peptides involved in posttranslational protein modification. Although the primary sequence of SUMO is substantially different from ubiquitin, its three-dimensional structure is similar to that of ubiquitin [1]. Protein modification by covalently bound SUMO, referred to as SUMOylation, is crucial to facilitate a wide variety of cellular processes *in vivo*. Similar to the ubiquitin system, SUMOylation is processed through the E1, E2 and E3 enzymes, and SUMO is attached to the target site of substrate proteins: ψ-K-x-D/E motifs in which ψ is a hydrophobic amino acid residue followed by Lys, any residue (x) and Asp/Glu. In contrast to ubiquitination, which tends to promote the degradation of target proteins, SUMOylation affects many aspects of the target proteins, for example, the subcellular localisation, protein stability, activity of transcriptional regulators and protein-protein interactions [2].

**Figure 1:**
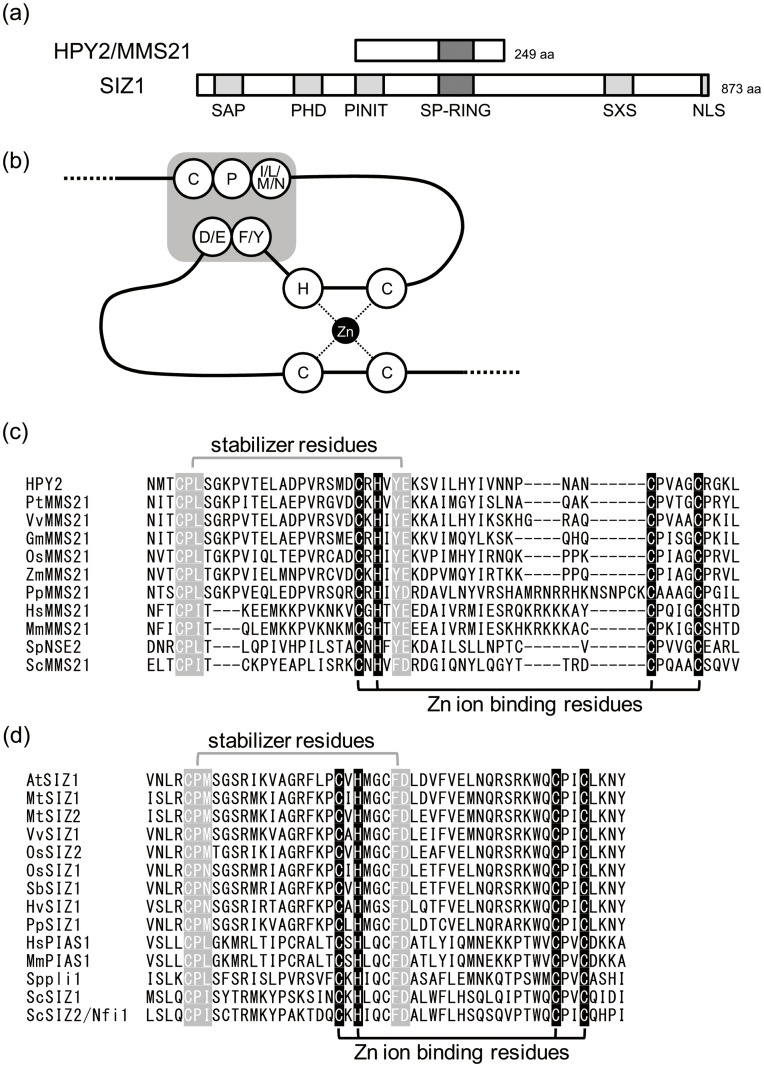
The SP-RING domain is conserved in plant SUMO E3 ligases. (a) The plant SUMO E3 ligases, HPY2/MMS21 and SIZ1. Characteristic domains are shown by grey boxes. SIZ1 possesses several domains, such as SAP, PHD, PINIT, SP-RING, SXS [4], while HPY2 possesses only SP-RING domain. (b) A schematic model of the SP-RING domain. The open circles indicate conserved amino acid residues, and the grey box marks the stabilising motif. A Zn ion coordinated in the SP-RING domain is indicated by a closed circle. (c) Sequence alignment of the SP-RING domain in the MMS21 homologues. The sequence data for the plant MMS21 homologues were obtained from the NCBI protein database and aligned based on Miura et al. (2007). *Vitis vinifera*, VvMMS21 (XP_002282690.1); *Populus trichocarpa*, PtMMS21 (XP_002317010.1); *Glycine max*, GmMMS21 (ACU20283.1); *Oryza sativa*, OsMMS21 (EEE64695.1); *Zea mays*, ZmMMS21 (ACF80287.1); *Physcomitrella patens*, PpMMS21 (XP_001767320.1). (d) Sequence alignment of the SP-RING domain in the SIZ1 homologues. The sequence data for the plant SIZ1 homologues were obtained from the NCBI protein database and aligned based on Miura et al. (2007). *Medicago truncatula*, MtSIZ1 (XP_003606454.1) and MtSIZ2 (ABD33066.1); *Vitis vinifera*, VvSIZ1 (XP_002282690.1); *Oryza sativa*, OsSIZ2 (NP_001051092.1) and OsSIZ1 (NP_001054517.1); *Sorghum bicolor*, SbSIZ1 (XP_002439205.1); *Hordeum vulgare*, HvSIZ1 (BAJ98904.1); *Physcomitrella patens*, PpSIZ1 (P_001767531.1).

The SUMO-mediated posttranslational modification is conserved among eukaryotes. Recent genomic analysis has revealed the presence of core SUMOylation enzymes, small subunits (SAE1a and SAE1b) and a large subunit (SAE2) of an E1 activation enzyme, an E2 conjugation enzyme (SCE1) and two E3 ligases (HPY2 and SIZ1) in Arabidopsis [3], [4], [5]. One of the most characteristic features of the plant SUMOylation system is that in plants only two types of E3 ligases have been characterised so far whereas four or more E3 ligases have been discovered in yeast and animals [6].

Despite the relative simplicity of the system, the biological functions of SUMO in plants appear to be very diverse. SIZ1 has been reported to function as a mediator of multiple environmental stimuli. For example, SIZ1 modulates the expression of several genes that respond to phosphate starvation or low temperature [7], [8]. In addition, SIZ1 regulates several heat shock proteins and heat shock transcription factors under high temperature and it also up- or down-regulates a wide variety of genes that respond to drought stress [9], [10]. SIZ1 is also known to modify the activity of abscisic acid (ABA)-insensitive 5 (ABI5), a transcription factor involved in ABA signalling [11]. The accumulated SA in the *siz1* mutants disturbs both the innate immunity response and flowering time [12], [13], suggesting that SIZ1 is involved in the homeostasis of SA. Recent studies have also reported that SIZ1 participates in copper accumulation and/or the response to copper and in the regulation of nitrogen assimilation [14], [15]. In contrast, HPY2 functions primarily in development by regulating cell proliferation in the meristem [16], [17]. The plant hormone auxin is known as a developmental signal that regulates the transition from the mitotic cell cycle to the endoreduplication cycle [18], and HPY2 acts downstream of the auxin pathway to promote cell proliferation [16], [19], [20].

In this study, we characterised the structural features of SUMO E3 ligases based on the recently analysed three-dimensional structure of the SP-RING domains [21] and found that both HPY2 and SIZ1 possess two conserved motifs in the SP-RING domain. Although both of the loss-of-function mutants display dwarf phenotypes, we found that the expression patterns of HPY2 and SIZ1 are distinct *in planta*. Consistently, the accumulation of SA induced dwarfism in *siz1-2* [13], whereas the loss of cell proliferation in the *hpy2-1* mutants is independent of SA, suggesting that HPY2 and SIZ1 contribute to plant growth and development through different mechanisms. We also show that the ectopic expression of SIZ1 driven by the *HPY2* promoter fails to rescue the dwarf phenotype in *hpy2-1*, further supporting the functional diversity of HPY2 and SIZ1. Finally, our genetic analysis demonstrates that the loss of these two E3 ligases in the *hpy2-1 siz1-2* double mutant causes lethality in embryogenesis, highlighting the notion that the SUMOylation mediated by these two E3 ligases is essential for early plant development.

**Figure 2:**
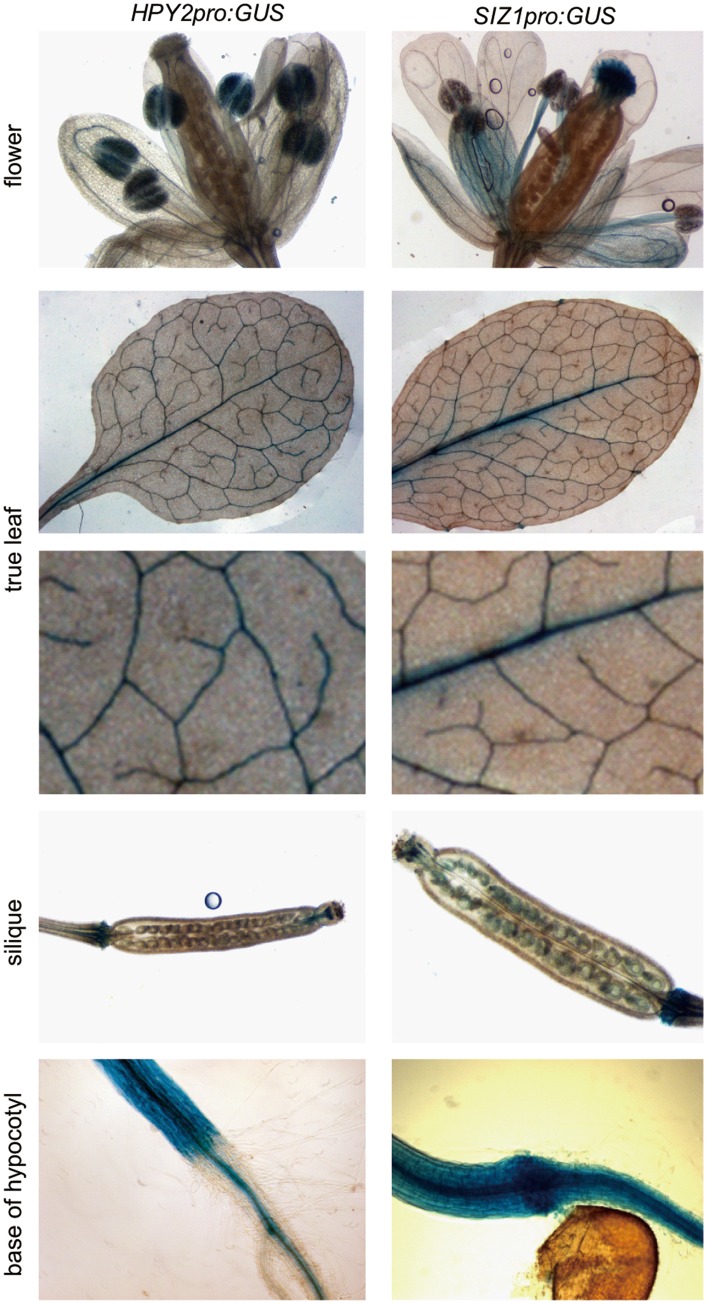
The expression pattern of *HPY2* and *SIZ1 in planta*. The promoter activity of *HPY2* and *SIZ1* in aerial tissues as visualised by the GUS activity in *HPY2pro:GUS* and *SIZpro:GUS* plants. The *HPY2* promoter activity is detected in the anther, leaf vein and hypocotyl cells while the *SIZ1* promoter activity is detected in stigma, leaf vein, young seeds and hypocotyl cells. The images for true leaves (upper panel) are magnified by ∼4-fold (lower panel) to show clear GUS signals in leaf veins.

## Results

### Plant SUMO E3 Ligases Possess Conserved SP-RING Domains

The most characteristic feature of the SUMO E3 ligases is the motif named the SP-RING domain, which is central for the interactions with both E2 and substrates [22]. As opposed to the RING-type ubiquitin E3 ligases, which contain two zinc ions held by seven cysteine residues and one histidine residue, the SP-RING domain contains one zinc ion in the similar domain and possesses a set of residues that stabilise the domain structure by hydrogen bonding and van der Waals contacts [20] (Figure 1b). It was previously shown that the zinc-binding cysteine and histidine residues are well conserved in Arabidopsis HPY2 and SIZ1 [4] (Figure 1c and 1d), and the substitution of these zinc-binding cysteine and histidine residues in PML, yeast MMS21 or human MMS21 affects the E3 ligase activity *in vitro* and growth velocity *in vivo*, respectively [23], [24], [25]. Mutations at the identical position of Arabidopsis HPY2 also lead to reduced E3 ligase activity and biological function [15].

**Figure 3:**
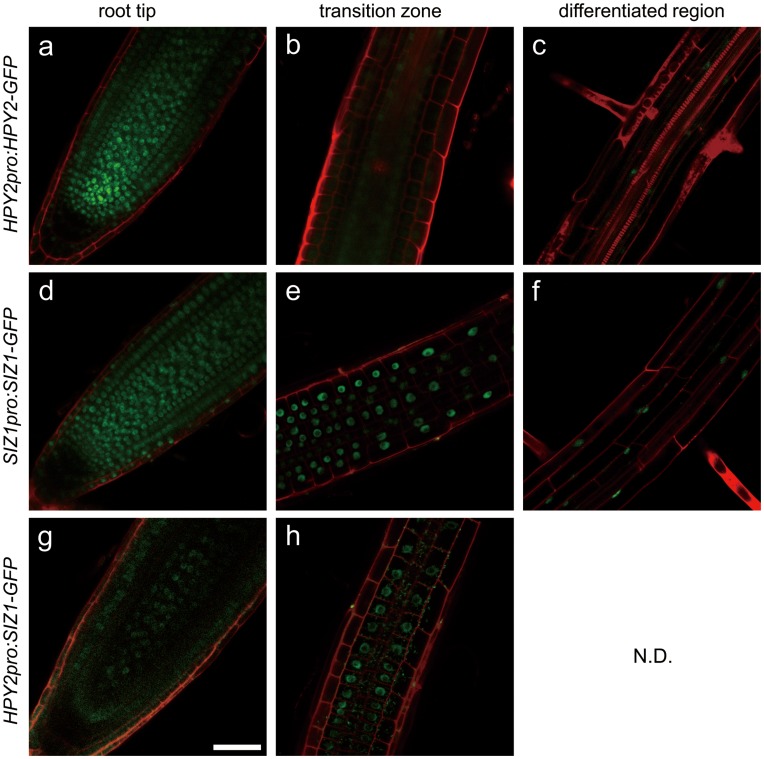
The protein localisation of HPY2 and SIZ1 in roots. Confocal microscopy of *HPY2pro:HPY2-GFP* (a–c), *SIZ1pro:SIZ1-GFP* (d–f) and *HPY2pro:SIZ1-GFP* (g, h) roots at the tip, transition zone and differentiated region. Bar = 50 µm.

We also mapped other stabilising residues in the SP-RING domains of HPY2 and SIZ1 and found that most of the responsible residues are also well conserved (Figure 1c and 1d). In HPY2, Cys159-Pro160-Leu161 and Tyr182-Glu183 may be responsible for the stabilisation, as the former is conserved among all of the MMS21-type E3 ligases we examined; the latter is also conserved, with the exception of ScMMS21, which possesses SIZ/PIAS-type Phe-Asp residues at the equivalent positions. As for SIZ1, the putative stabilising motifs, Cys363-Pro364-Met365 and Phe385-Asp386, are well conserved, though Met365 is replaced by a Leu or Ile residue in yeast and animal SIZ1. In addition to this loose conservation, several SIZ proteins in monocots possess Asn at this position (Met365), suggesting that this residue may be relatively flexible [26] (Figure 1d). Although the crystal structure of the plant SP-RING domain is not yet available, these functional annotations suggest that the specific features of SUMO E3 ligases are conserved in plants. Other than the SP-RING domain, HPY2 does not possess any obvious functional domains previously identified while SIZ1 contains other domains, such as SAP, PHD, PINIT and SXS, that are characteristic to PIAS/SIZ proteins [27] (Figure 1a).

### SIZ1 and HPY2 Exhibit Different Expression Patterns in Planta

Both HPY2 and SIZ1 are predominantly localised in nuclei [8], [16], which is consistent with the previous reports that most of the SUMO targets in plants are nuclear proteins [6]. To examine the expression patterns of HPY2 and SIZ1 in aerial tissues, we generated transgenic plants carrying the constructs *HPY2pro:GUS* and *SIZ1pro:GUS*. As shown in Figure 2, we found that the *HPY2* promoter is active in the anther, vein and hypocotyl cells, whereas the *SIZ1* promoter is active in stigma, vein, young seed and hypocotyl cells. These data are largely consistent with previous observations that the expression of *HPY2* is more restricted compared to that of *SIZ1* [8], [9], [16] To observe the accumulation of HPY2 and SIZ1 in the roots, we used transgenic plants carrying the constructs *HPY2pro:HPY2-GFP* and *SIZ1pro:SIZ1-GFP*, as it was previously shown that each of these GFP constructs encodes a functional fusion protein that rescues their respective original mutations [12], [16]. The SIZ1-GFP proteins are expressed in all cells, including the proliferating cells at the root tip and the more distal cells that are fully differentiated. As previously described, HPY2-GFP expression is relatively strong in the proliferating cells at the meristem, and we detected weak HPY2-GFP signals only in the vascular cells of mature roots (Figure 3a–f). These data show that HPY2 and SIZ1 are expressed in partially overlapping cells but that they also display distinct expression patterns.

**Figure 4:**
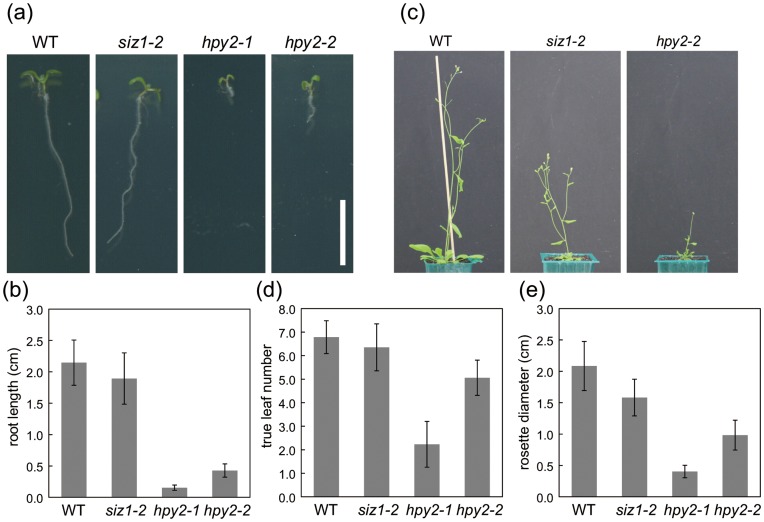
The dwarf phenotypes of SUMO E3 ligase mutants. (a) Seven-day-old wild-type (WT), *siz1-2*, *hpy2-1* and *hpy2-2* seedlings. (b) Quantification of the root length of seven-day-old seedlings. (c) Twenty five-day-old WT, *siz1-2* and *hpy2-2* plants. Most of the *hpy2-1* plants are no longer viable after bolting. (d) Quantification of the true leaf number of fifteen-day-old seedlings. (e) Quantification of the rosette diameter of fifteen-day-old seedlings. Bar = 1 cm in (a).

### The siz1 and hpy2 Mutants Exhibit Distinct Dwarf Phenotypes

The Arabidopsis mutants of the two SUMO E3 ligases, *siz1* and *hpy2,* show growth defects [16], [28]; however, when examined closely, their phenotypes are not identical. The loss-of-function *siz1-2* mutants are indistinguishable from the wild-type at an early seedling stage, but they begin to represent strong growth retardation at the later vegetative to reproductive stages (Figure 4). These growth defects are pronounced under conditions that are unfavourable for the *siz1-2* plants, which can grow similar to the wild-type under moderate conditions, e.g., in the presence of phosphate or without excessive temperature or humidity stress, during the early vegetative stage [7]. In contrast, the *hpy2-1* mutants already display severe growth defects just after germination due to the lower cell cycle activity and collapsed meristem structure; for example, they develop roots only <10% the length of the wild-type [16] (Figure 4a, b). These growth retardation phenotypes are similar to those of the *hpy2-2* mutants, which are relatively mild in comparison [16], [17] (Figure 4a, b). The *hpy2-1* mutants often terminate their growth before bolting, whereas the *hpy2-2* and *siz1-2* mutants survive through the vegetative stage and bolt. At the reproductive stages, the *siz1-2* plants develop smaller leaves and shorter stems than the wild-type plants, yet they still propagate (Figure 4c). In contrast, the *hpy2-2* plants that survive through the seedling stage develop extremely small leaves and fasciated stems [16] (Figure 4c). By quantifying the number of leaves and the diameter of rosette leaves at 15 days after germination, we found that shoot development is severely retarded in *hpy2-2* (Figure 4d, e).

**Figure 5:**
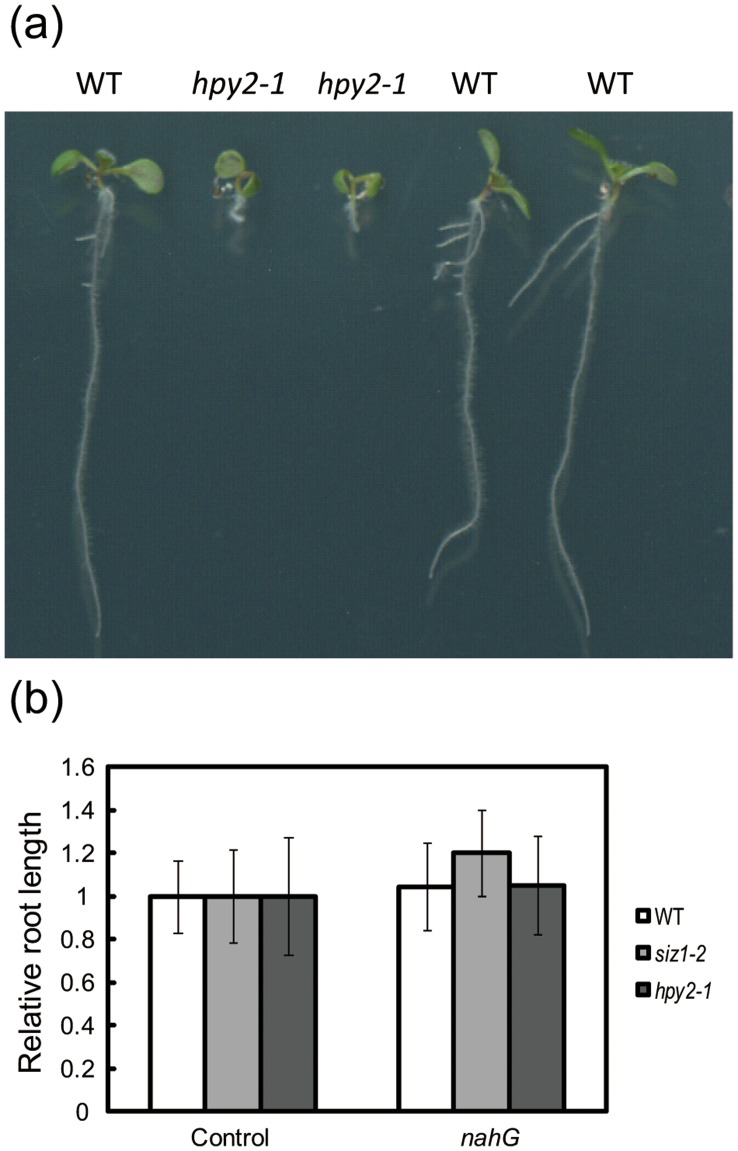
Introduction of *nahG* in *hpy2-1* does not restore the dwarf phenotype. (a) Seven-day-old WT and *hpy2-1* seedlings carrying *nahG*. (b) Relative root length of WT, *siz1-2* and *hpy2-1* plants with or without *nahG*.

### The Dwarf Phenotype of hyp2-1 is not Caused by SA Accumulation

As previously reported, the loss of the SIZ1 function increases the SA content [13]. Furthermore, transgenic plants expressing SUM1 with or without the C-terminal Gly-Gly also show morphological defects caused by the accumulation of SA [29]. The removal of SA by the introduction of the bacterial enzyme nahG or the additional mutation in the SA biosynthesis pathway gene *SALICYLIC ACID INDUCTION DEFICIENT 2* (*SID2*) partially rescues the dwarf phenotypes of *siz1* or the dominant SUMO overexpresser, respectively [28], [29]. Although the SA content in *hpy2-1* or *hpy2-2* has never been directly measured, the *hpy* phenotypes appear different from the SA-dependent phenotypes of *siz1-2.* The *siz1-2* mutation causes dwarfism but the meristem organisation is largely indistinguishable from the wild type. (Figure S1) In contrast, the tissue initiation pattern is severely disturbed in the *hpy2* mutants. To test whether SA is responsible for the *hpy2-1* phenotype, we crossed *nahG* into the *hpy2-1* mutant and isolated F2 plants possessing the homozygous *nahG* transgene and the heterozygous *hpy2-1* mutation. In the F3 progeny, we found that approximately one quarter of the plants segregate for the dwarf phenotype and three quarters for wild-type-like healthy plants (Figure 5a). By genotyping, we confirmed that the presence of *nahG* does not cause visible effects on growth in *hpy2-1* because the *hpy2-1 nahG* seedlings barely survived during the vegetative stage, similar to the *hpy2-1* mutants (Figure 5b). These results suggest that the developmental defects in the *hpy2-1* mutants are not caused by SA accumulation and that these SUMO E3 ligases may act independently during plant development.

### Reciprocal Expression of SIZ1 and HPY2 does not Complement the Single-mutant Phenotypes

**Figure 6:**
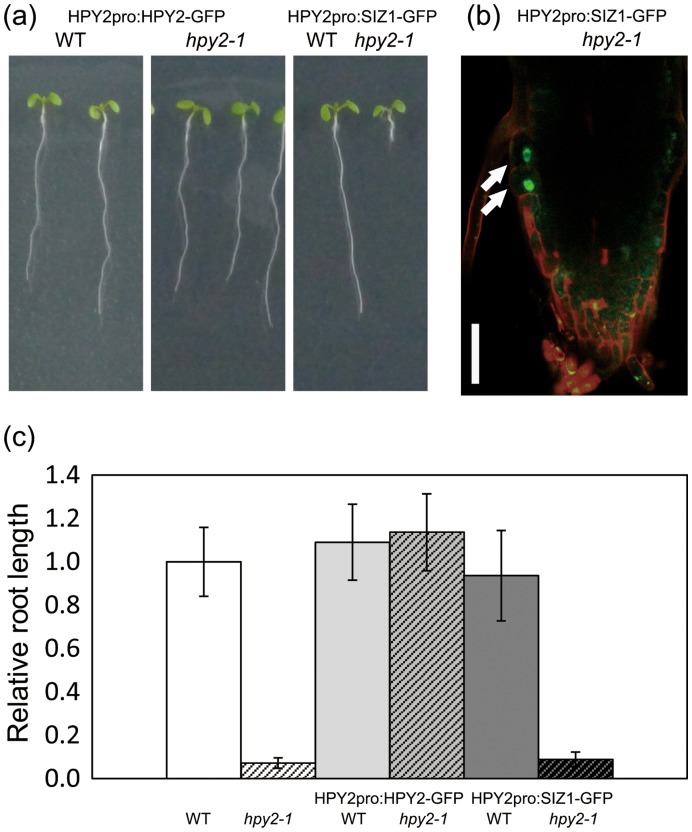
Ectopic expression of *SIZ1* by the *HPY2* promoter does not rescue the *hpy2-1* phenotype. (a) Seven-day-old WT and *hpy2-1* seedlings carrying *HPY2pro:HPY2-GFP* or *HPY2pro:SIZ1-GFP*. (b) Microscopic observation of *HPY2pro:SIZ1-GFP* in *hpy2-1* root tips. Despite the expression of SIZ1-GFP fusion proteins, as indicated by white arrows, the meristem defects are not restored in *hpy2-1*. (c) Relative root length of WT, *hpy2-1*, HPY2p:HPY2-GFP in WT, HPY2p:HPY2-GFP in *hpy2-1*, HPY2p:SIZ1-GFP in WT and HPY2p:SIZ1-GFP in *hpy2-1*.Bar = 50 µm in (b).

To explore the functional relationship between HPY2 and SIZ1 further, we ectopically expressed the functional SIZ1-GFP proteins in *hpy2-1* and tested whether SIZ1 can complement the dwarf phenotype of *hpy2-1.* To drive the expression of SIZ1-GFP in *hpy2-1,* we used the 2.2 kb upstream sequence of *HPY2*, which includes the sequence used for the construction of the *HPY2pro:HPY2-GFP* constructs. We have previously shown that this construct complements the *hpy2-1* mutant phenotype [16] (Figure 6a). Using more than 25 independent transgenic plants, we confirmed the expression of the SIZ1-GFP proteins in the root meristem and their nuclear localisation (Figure 3g, h). However, we found that the transgenic *hpy2-1* plants harbouring the *HPY2pro:SIZ1-GFP* constructs display a similar dwarfism as *hpy2-1* (Figure 6a). We quantified the root length of wild-type, *hpy2-1* and *hpy2-1* carrying *HPY2pro:SIZ1-GFP* but we did not find significant difference between *hpy2-1* and *hpy2-1* carrying *HPY2pro:SIZ1-GFP* (Figure 6b), suggesting that SIZ1 does not replace the *in vivo* functions of HPY2.

To test whether HPY2 complements *siz1*-2, we expressed the *HPY2* cDNA under the control of cassava virus (CsV) promoter, which drives gene expression throughout the entire plant [30]. Similar to *siz1-2*, the transgenic plants expressing *HPY2* in the *siz1-2* background still display short stems and small rosette leaves (Figure S2), suggesting that the ubiquitous expression of *HPY2* in the *siz1-2* mutants does not rescue their growth defects.

### Double Mutants of hpy2-1 and siz1-2 Display Embryonic Lethality

**Figure 7:**
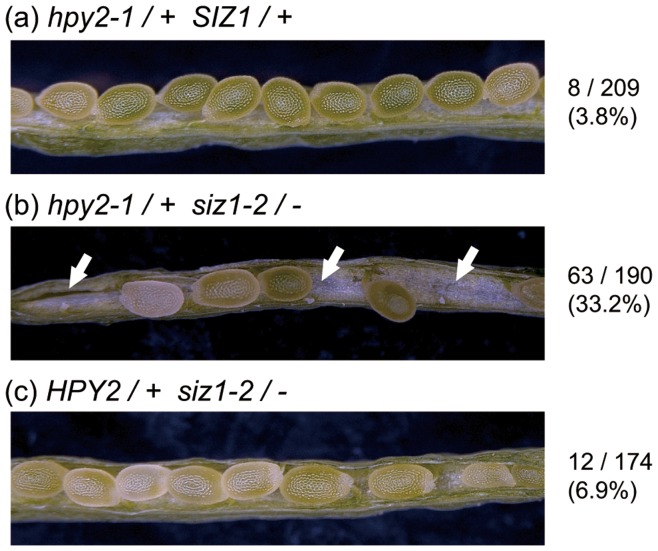
Double mutants of *hpy2-1* and *siz1-2* are embryonic lethal. Maturing seeds in the siliques of *hpy2-1*/+ (a), *hpy2-1*/+ *siz1-2*/− (b) and *siz1-2*/− (c). White arrows in (b) mark collapsed seeds. Numbers on the right represent the percentage of aborted seeds.

The loss of SUMOylation by the *sae2* or *sce1* mutation causes embryonic lethality [31], indicating that SUMO plays crucial roles during embryogenesis. These data are consistent with other reports that the double mutation of AtSUMO1 and AtSUMO2, the major contributors of the SUMO function in Arabidopsis, is also lethal at an embryonic stage [31]. Based on these observations, we hypothesised that, if HPY2 and SIZ1 are the major E3 ligases in plants, their concomitant loss in the respective double mutant would lead to a similar lethality. To test this hypothesis, we crossed *hpy2-1* and *siz1-2* and screened the F2 generation for the double mutant. As predicted, however, we did not recover the double mutants among over 50 seedlings we genotyped. We then tested the segregation of F3 population derived from the *hpy2-1*/+ *siz1-2*/− parental lines. Among the 126 seedlings we tested, 40 and 86 lines carried the wild-type or heterozygous *HPY2* locus, respectively, but we did not detect any plants homozygous for *hpy2-1*. These results strongly suggest that the *hpy2-1 siz1-2* double mutant is embryonic lethal. Consistently, we found that 33.2% of the seeds in the *hpy2-1*/+ *siz1-2*/− siliques are collapsed (Figure 7), suggesting that these seeds may represent the *hpy2-1 siz1-2* double mutants. A recent study has revealed that SIZ1 is required for gametogenesis and *siz1-2* seeds are frequently collapsed under conditions of low nitrogen [15], [32]. Although we also found several collapsed seeds in the *siz1-2* siliques, the frequency was very low (6.9%) and we observed strong enhancement of seed abortion by the *hpy2-1* mutation (Figure 7).

## Discussion

### SUMOylation is an Essential Posttranslational Modification System in Eukaryotes

HPY2 and SIZ1 are structurally conserved SUMO E3 ligases in Arabidopsis and mediate cellular processes through the SP-RING domain. Two recently published studies on the SP-RING structure have revealed that this domain contains two characteristic motifs: one that coordinates a zinc ion with cysteine and histidine residues and another with conserved residues facing each other to stabilise the SP-RING structure [21], [33]. Our sequence analysis clearly shows that both of these important motifs are well conserved in the plant kingdom from lower to higher plants.

In Arabidopsis, only two SUMO E3 ligases, MMS21-like HPY2 and SIZ1, have been analysed to date, and our data show that the SUMOylation mediated by these two E3 ligases is essential for early developmental processes. Given that both of these E3 ligases are expressed in various post-embryonic tissues, it is likely that they also play major roles in the growth and development of post-embryonic organs. Other genes are predicted to be potential SUMO E3 ligases because they possess the features of the SP-RING domain [27], but it remains unknown whether they have any functional roles *in vivo*. This situation contrasts with animals in which other types of E3 ligases, such as the HECT-like SUMO E3 ligase RanBP2, are also identified [34]. In budding yeast, at least three different SP-RING-type SUMO E3 ligases, ScSIZ1, ScSIZ2 and ScMMS21, have been shown to SUMOylate certain specific and common substrates [25]. Interestingly, the combination of severe *scmms21* mutant alleles and *scsiz1* or *scsiz2* mutations is not viable, and weak mutant alleles of *mms21* with a double *scsiz1 scsiz2* mutation are also lethal [25]. These results support the view that SUMO is involved in fundamental process(es) in eukaryotic cells and that MMS21- and SIZ-type SUMO E3 ligases together have vital functions for survival.

### Functional Diversity of SUMO E3 Ligases in Plants

Our genetic analysis demonstrates that the double mutant of *hpy2-1* and *siz1-2* is embryonic lethal. Although the exact cellular functions of HPY2 and SIZ1 are not established, we predict that these two SUMO E3 ligases play essential but most likely distinct roles during embryonic and post-embryonic development because the ectopic expression of SIZ1 under the control of the *HPY2* promoter does not restore the *hpy2-1* phenotype. We also show that, unlike *siz1-2*, the developmental abnormalities in the *hpy2-1* mutants do not result from SA accumulation, supporting the view that HPY2 and SIZ1 function through different pathways.

Both HPY2 and SIZ1 can use AtSUMO1 *in vitro* but whether they SUMOylate same substrates is currently unclear. Recently, several researcher groups have established proteomic approaches to identify SUMO targets and have isolated more than a hundred Arabidopsis proteins as putative SUMO substrates *in vivo* or *in vitro* [5], [35]. This large number of substrates implies the requirement of E3 activities in diverse cellular processes. Elucidating the specific or common targets of HPY2 and SIZ1 to clarify their functional diversity during plant development will be one of the most important tasks in future studies.

## Materials and Methods

### Plant Materials and Growth Conditions

The Arabidopsis mutants and transgenic lines used in this study were described previously [8], [12], [16], [28]. All of the lines were in the Columbia (Col) background. The seeds were surface-sterilised in 70% ethanol for 1 min, then in 20% (v/v) sodium hypochlorite for 5 min, and rinsed three times in sterile water. The sterilised seeds were plated on Murashige and Skoog (MS) medium supplemented with 1% (w/v) sucrose and 0.5% Gelrite. After a cold treatment in the dark for 1 day, the plates were placed vertically and incubated under continuous light at 22°C in an MLR-351 growth chamber (SANYO).

### Microscopy

The roots were visualised using seedlings stained with 10 mg/ml propidium iodide (PI). Fluorescence microscopy was performed using a Carl Zeiss LSM700 confocal laser microscope. To observe the seeds in the siliques, fully grown siliques are harvested and peeled using a dissecting microscope. The micrographs of the seeds were captured using a Leica MZ16FA microscope.

### Histochemical Analysis of GUS Activity

The promoter region of *SIZ1* (-2,035 to -7 from the ATG [36]) or *HPY2* (-2,058 to -59 from the ATG) was introduced into pCambia1391Z. The resulting constructs were transformed into Arabidopsis, and hygromycin-resistant plants were obtained. The transgenic plants were incubated for 4 h at 37°C in GUS reaction buffer (1.9 mM 5-bromo-4-chloro-3-indolyl-β-D-glucuronide, 0.5 mM K_3_Fe(CN)_6_, 0.5 mM K_4_Fe(CN)_6_, 0.3% (v/v) Triton X-100, 20% (v/v) methanol and 10 mM EDTA in 100 mM sodium phosphate, pH 7.0). The stained seedlings and tissues were washed with 70% (v/v) ethanol four times to stop the reaction and remove the chlorophyll. Representative seedlings were photographed using Normarski optics and a DM RXA-6 or MZ-FLIII microscope (Leica).

### Construction of the HPY2pro:SIZ1-GFP Vectors

The 2.2-kb upstream sequence of *HPY2* was PCR amplified from the Arabidopsis genome using primers Xba1-MMS21pro-F (5′-GCCTGCAGGTCGACTCTAGATGAATTGAAGCAATGCTAC-3′) and gMMS21-proR (5′-CATATCTATCGCTCCTTCGCTCC-3′). The 3.3-kb *SIZ1-GFP* fragment was PCR amplified using primers MMS21-IF-SIZ1-cDNA-F (5′-GAAGGAGCGATAGATATGGATTTGGAAGCTAATTG-3′) and Sac1-GFP-R (5′-CGATCGGGGAAATTCGAGCTCCTGGTCACCAATTCACACG-3′) from the *SIZ1pro:SIZ1-GFP* construct [11]. Both of the fragments were cloned using the In-Fusion Advantage PCR cloning kit (Clontech) into the pGWB601 vector digested with Xba1 and Sac1 [37]; the underlined primer sequences were responsible for the recombination between the PCR products and the digested pGWB601 vector. The resulting vectors were transformed into *hpy2-1* heterozygous plants using an Agrobacterium-mediated floral dip method. The transformants were screened on medium containing 10 mg/l glufosinate-ammonium (SIGMA).

## Supporting Information

Figure S1 **The root tip organisation of** ***siz1-2*** **.** Confocal microscopy of wild-type and *siz1-2* roots. Bar = 50 µm.
(TIF)

Figure S2 **Ectopic expression of** ***HPY2*** **by the cassava virus (CsV) promoter does not rescue the** ***siz1-2*** **phenotype.** (a) 30-day-old wild-type, *siz1-2*, *CsVpro:HPY2* in *siz1-2* and *CsVpro:HPY2* in wild-type. (b) RT-PCR analysis of CsV promoter driven *HPY2* cDNA and endogenous *SIZ1* cDNA. A diagram representing the *CsVpro:HPY2* vector and the region used for RT-PCR.
(TIF)
