# Supplementary figures and images for: MMS21/HPY2 and SIZ1, Two Arabidopsis SUMO E3 Ligases, Have Distinct Functions in Development
Source: PLoS One. 2012 Oct 8;7(10):e46897. doi: 10.1371/journal.pone.0046897 (PMC3466189; doi:10.1371/journal.pone.0046897)

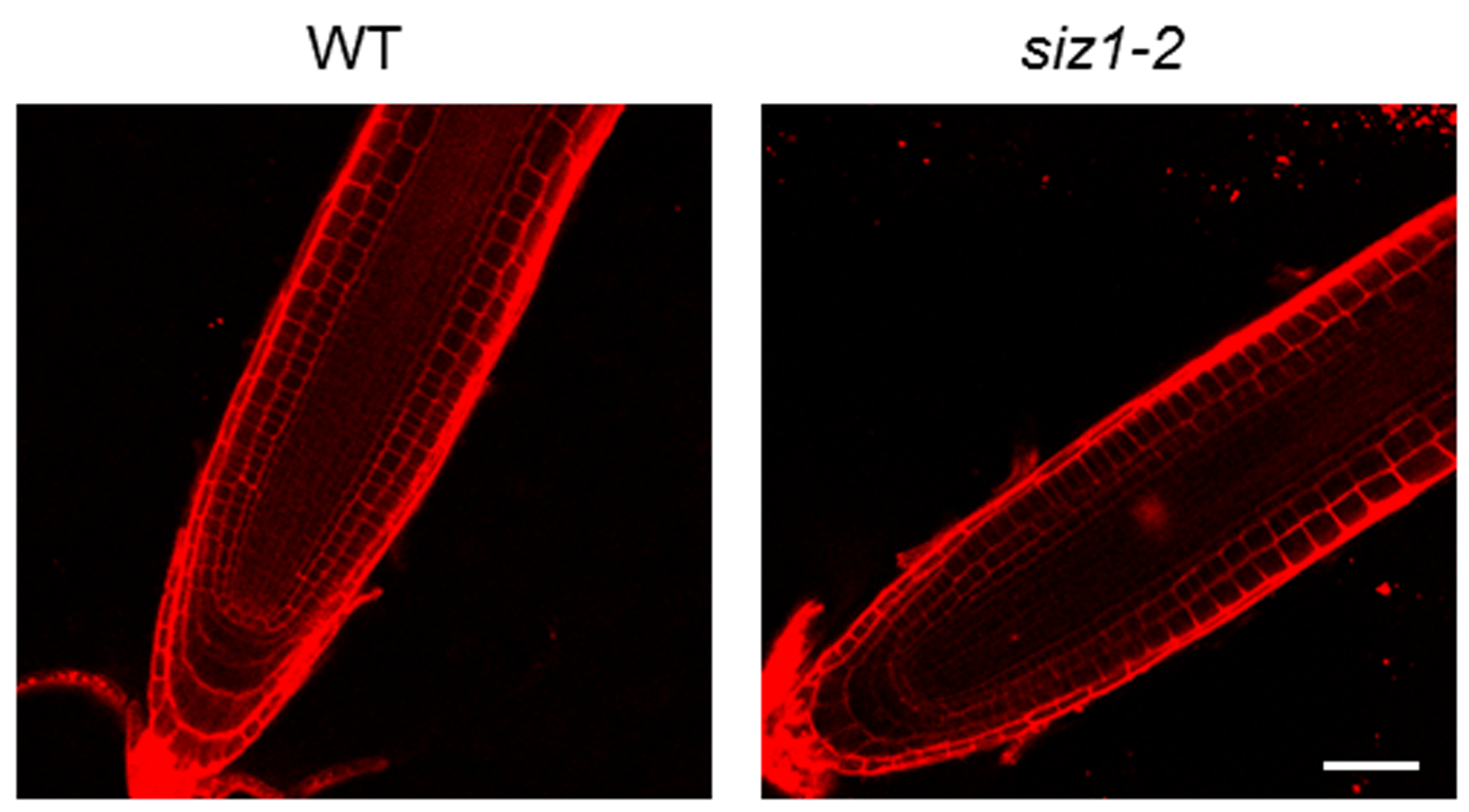

Supplement: Figure S1 — The root tip organisation of siz1-2 . Confocal microscopy of wild-type and siz1-2 roots. Bar = 50 µm. (TIF) [file pone.0046897.s001.tif]

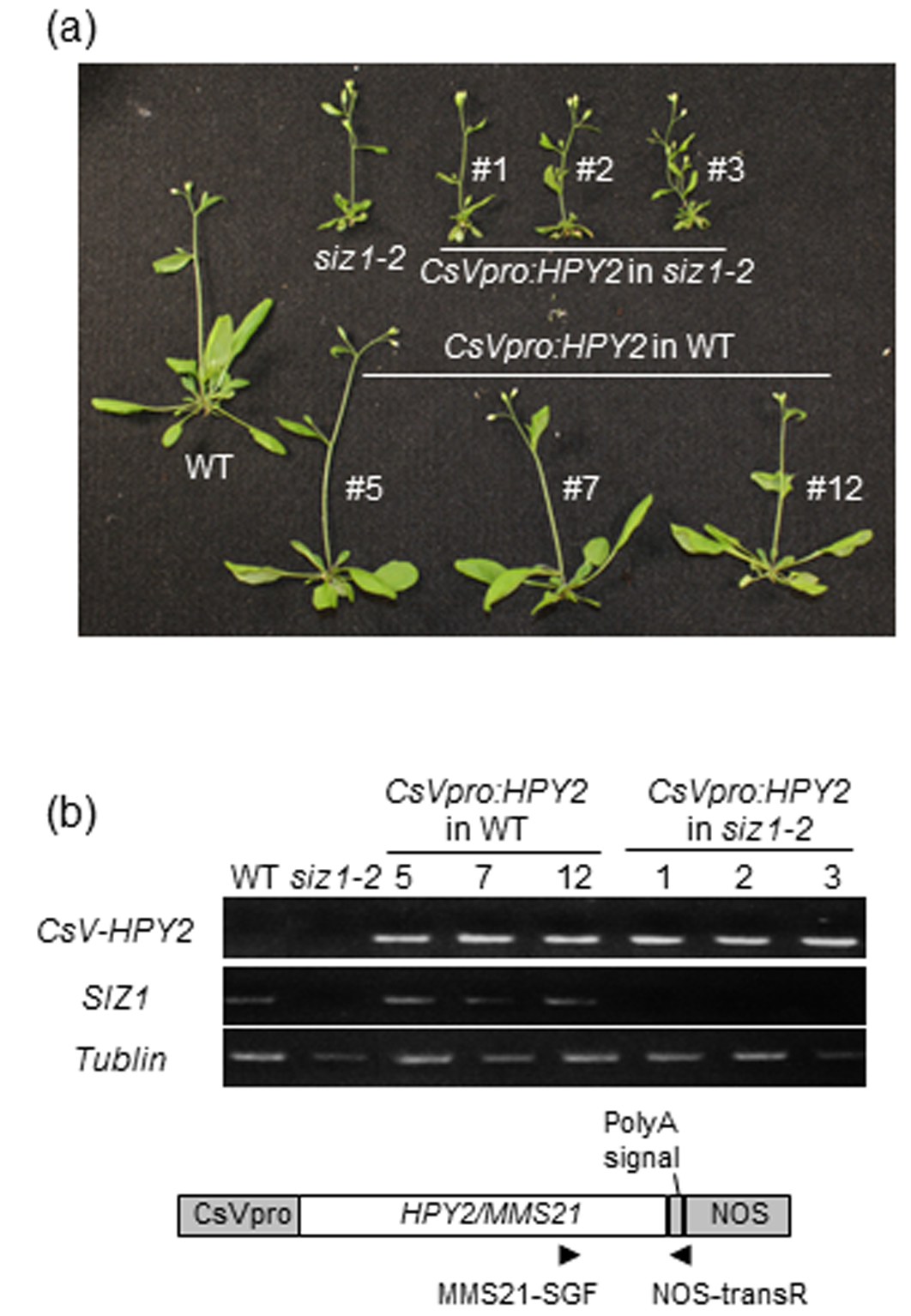

Supplement: Figure S2 — Ectopic expression of HPY2 by the cassava virus (CsV) promoter does not rescue the siz1-2 phenotype. (a) 30-day-old wild-type, siz1-2, CsVpro:HPY2 in siz1-2 and CsVpro:HPY2 in wild-type. (b) RT-PCR analysis of CsV promoter driven HPY2 cDNA and endogenous SIZ1 cDNA. A diagram representing the CsVpro:HPY2 vector and the region used for RT-PCR. (TIF) [file pone.0046897.s002.tif]
